# A Novel Mutation in Aspartoacylase Gene; Canavan Disease

**Published:** 2015

**Authors:** Mahmoudreza ASHRAFI, Alireza TAVASOLI, Pegah KATIBEH, Omid ARYANI, Mohammad VAFAEE-SHAHI

**Affiliations:** 1 Pediatric Neurology Division, Growth and Development Research Center, Children’s Medical Center, Pediatric Center of Excellence, Tehran University of Medical Science, Tehran, Iran; 2 Department of Medical Genetic, National Institute for Genetic Engineering and Biotechnology, Tehran, Iran

**Keywords:** Canavan disease, Aspartoacylase deficiency, Aspartoacylase enzyme, ASPA gene

## Abstract

**Objective**

Canavan disease (CD) is a type of vacuolating leukodystrophy with autosomal recessive inheritance. Aspartoacylase deficiency results in decrease of myelin biosynthesis, dysmyelination and brain edema. Although CD is a very common in Ashkenazi Jews patients, several cases have been reported from non-Jewish population. This report is based on a homozygous C.202G>A mutation in the ASPA gene identified from an Iranian patient. To our knowledge, this type of mutation has not been reported in non-Jewish population in the literature.

## Introduction

Canavan disease (CD) is a type of vacuolating leukodystrophy with autosomal recessive inheritance (1). This neurodegenerative disorder is caused by a defect in the aspartoacylase (ASPA) gene encoding the critical enzyme aspartoacylase, which has the role of hydrolyzing N-acetyl-L-aspartic acid (NAA) and providing the acetyl group to oligodendrocytes for myelin synthesis (2, 3). Aspartoacylase deficiency results in the reduction of myelin biosynthesis, dysmyelination and brain edema (4). Although CD is very common in Ashkenazi Jews, several cases have also been reported from non-Jewish population (4). To date mutation analysis on ASPA gene among Jewish and non-Jewish patients has revealed more than 70 different types of mutations (2). E285A (p.Glu285Ala) and Y231X (p.Tyr231X) mutations are the most common types of mutations identified among Ashkenazi Jews (more than 98% of reported mutations) while A305E (p.Ala305Glu) is the most common among non-Ashkenazi Jewish population (about 30-60% of reported mutations) (5, 6). This report is based on a homozygous C.202G>A mutation in the ASPA gene of an Iranian patient. To our knowledge, this mutation was not reported in non-Jewish population in Iran.

## Case presentation

**Fig 1:**
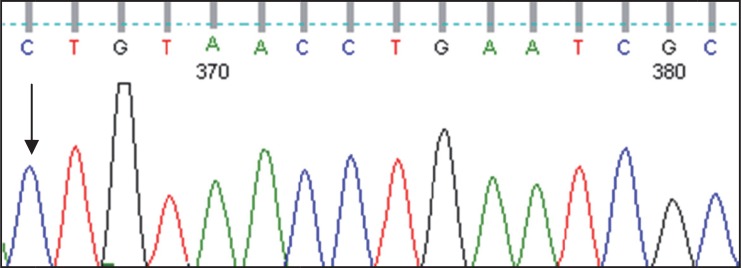
Homozygous mutation as C.202G>A

This report is on a 10 months old male infant who was born through normal vaginal delivery with birth weight of 2900 grams (gm), head circumference of 33 centimeters (cm) and height of 49.5 cm. He was the first child of consanguineous parents (first cousin) and his mother had no previous history of abortion or dead fetus. The child was nearly normal until the age of 3 months when he gradually developed lethargy, poor feeding, and inability to hold his neck with progressive increase in his head circumference. On physical exam at the age of 10 months, the child had poor weight gain (weight: 6.6 kg), large (48 cm) head circumference (above 95% percentile), visual problem (poor eye contact and impaired fixing and following), and a sluggish response to sound along with poor feeding. Based on his mother’s statements the child had been floppy at the onset of the disease, but at the age of 10 months, he became spastic with bilateral extensor plantar response and inability to sit. The patient was investigated for possible neurodegenerative disease and metabolic work-ups such as serum ammonia, lactate, pyruvate, blood gas, tandem mass metabolic screening (MS/MS), urine organic acid profile and plasma amino acids profile through HPLC method were requested. All the findings were normal except for an increased N-Acetyl-L-Aspartic Acid in urine. However, an audio metric brainstem response (ABR) and visual evoked potential (VEP) tests have shown abnormal findings. Magnetic resonance imaging (MRI) of the brain revealed diffuse symmetrical white matter of centrum semiovale and involvement of subcortical U fibers, along with some degree of hyper intensity in the thalamus, basal ganglia, periventricular white matter, brainstem and cerebellum. Because of the suspicion of Canavan disease on physical examination, further laboratory investigation, brain imaging and the Aspartoacylase enzyme activity test on the skin culture fibroblast were found in favor of the Canavan disease. ASPA-specific genomic DNA fragments were amplified by PCR method using specific sets of primers (Table 1). DNA sample from the patient was investigated by sequencing method. A homozygous missense mutation as C.202G>A in exon 1 was found (Figure1). This finding has not been reported in literatures. The parent’s DNA investigation was not done due to refusal of the parents.

**Table1:** List of primers that have been used for genetic study in our patient

Primer name	Sequence
ASPA-1F	GCAGGGCTAAAGAAGGG
ASPA-1R	CATACGACTGCATGTACGG
ASPA-2F	TGACCAGCCACATAAATGCAC
SPA-2R	GCCTGGCTATGGAATAAACCC
SPA-3F	TTTTTGATCATGGTTCTGG
ASPA-3R	CAAAAATTACAGGGTGGC
ASPA-4F	TTCTGCTTCACGTTTTGC
ASPA-4R	TGTCTATCCTGGCCATTG
ASPA-5F	TGGAGTGCAATGGCTTACTG
ASPA-5R	AGCGTGCAGGCCATACTTAC
ASPA-6F	TCAGATCACTTGCCTGCATC
ASPA-6R	TGCCTACCGAATAAGGCAC

## Discussion

Canavan disease (CD) is a rare vacuolating leukodystrophy that is caused by aspartoacylase deficiency (8). The substrate of this enzyme is NAA, which is exclusively synthesized in the brain. NAA is hydrolyzed by aspartoacylase to acetate that is necessary for myelin synthesis and aspartate (6, 9). Deficiency of aspartoacylase leads to the accumulation of NAA in the brain and pathologically results in spongy degeneration of the white matter (2, 9). Clinically, two types of CD have been described. The most common type of CD is the neonatal/infantile form that is more severe in clinical presentation compared to the juvenile type of the disease (5, 7). Clinical symptoms of neonatal/ infantile CD usually start between the age of 2 - 6 months and appears with lethargy, poor feeding, axial hypotonia, lack of neck holding in pull to sit maneuver, progressive hyperreflexia, spasticity along with macrocephaly and developmental regression. Cortical blindness and optic atrophy accompanies seizure in later stages (7). The clinical course of our patient was consistent with the infantile type of CD. Extraction of c-DNA and gene for human ASPA helps to describe the molecular basis of the CD. The only known gene for Canavan disease is ASPA gene that is localized on the short arm of chromosome 17(17p13-ter region) (5). Six exons and five introns are included in this gene (4). Investigation of mutations in patients with CD has disclosed missense, nonsense and splice-site mutations, deletions and or insertions (4). Canavan disease is seen commonly in Ashkenazi Jewish population but with less prevalence among other ethnicity (2, 5, 7). Two predominant mutations E285A and Y231X account for 98% of chromosomal mutations among Jewish population. A305E is the most prevalent mutation among Western Europe English, Dutch and German patients. The A305E missense mutation is responsible for 48% (24/50) of mutations in western European ancestry (10). Y231X (p.Tyr231X) and A305E (p.Ala305Glu) mutations result in complete loss of ASPA activity function. However, E285A (p.Glu285Ala) mutation has about 3% of wild type enzyme activity (5, 10). Various genetic mutations among non-Jewish patients have been reported. **Table 2 **presents some of these mutations in addition to the type of mutation identified in this study (7, 11, 12). Homozygous mutation as in the exon 1 of ASPA gene in the present case is a novel mutation that was not reported in literatures. The severity of brain involvement in current study does not seem to be different from the other mutations while it was relatively severe. Cultured skin fibroblasts are required to confirm ASPA deficiency and to determine carrier state or disease in high-risk individuals. The cultured skin fibroblasts of the patient in our study also confirmed that the patient had ASPA deficiency. Had the mutation that caused the CD been specified in the family, it would make prenatal diagnosis possible and to recommend parents with such disorder to plan for the next pregnancy.
